# Post-COVID-19 headache- NDPH phenotype: a systematic review of case reports

**DOI:** 10.3389/fpain.2024.1376506

**Published:** 2024-05-14

**Authors:** Neetu Rani Dhiman, Deepika Joshi, Royana Singh, vyom Gyanpuri, Anand Kumar

**Affiliations:** Department of Neurology, Banaras Hindu University, Varanasi, India

**Keywords:** COVID-19, pain, headache—classification, review—systematic, persistent (chronic) pain

## Abstract

**Background and objectives:**

Post-acute COVID-19 syndrome or “long COVID” affects patients even after the recovery from Covid infection in various ways. Persistent headache or New Daily Persistent Headache (NDPH) is one of such symptoms. In this review, we will discuss about the case-reports of post covid-19 headache- NDPH phenotype both after and in the course of COVID-19 infection.

**Methods:**

Case reports/studies talked about patients having NDPH around the disease either immediately or late post COVID were included. Data was taken from the source and synthesised on a qualitative basis.

**Results:**

Literature search showed 3,538 articles, out of which 12 were screened as per the eligibility criteria and finally, 4 case reports on NDPH and Covid-19 were chosen for analysis from the database and by human search. All case reports justify the criteria for acceptability in quality for this systematic review.

**Conclusion:**

NDPH in and around Covid 19 infection is something that is currently an ingenious debated topic in the scientific community. More case studies should be written and published on the same subject so that a large systematic review could be conducted.

**Trial Registration Information:**

The review is registered in Prospero with no. **Identifier** (CRD42022354912).

**Systematic Review Registration:**

https://www.crd.york.ac.uk/, PROSPERO (CRD42022354912).

## 1 Introduction

COVID-19 is a viral infection in which headache is known to be an early symptom (1). The majority of individuals with a COVID-19-related headache describe it as diffuse, pressing, and of moderate to severe intensity. Individuals who had a history of COVID-19-related headache bouts were more likely to have comorbid conditions, main headache disorders, or dehydration (2) in a sizable portion of people, ranging from 14%–60% (3, 4).

NDPH is characterized by an abrupt or acute onset of headache followed by a daily, unremitting, and continuous course for more than 3 months (5, 6). Patients knowing the exact time of onset is a requirement for the diagnosis of NDPH onset, according to ICHD-3 (6). Twenty to eighty percent of patients can recall the day on which their headaches first appeared, and almost eighty percent can pinpoint the precise month. Between 0.03% and 0.1% of the population as a whole has NDPH (7, 8). Between 2.24% and 11% of people with chronic daily headaches have NDPH (9, 10). In youth who experience chronic daily headaches, it may reach 36% (11). NDPH is a diverse set of diseases with various pathophysiology. The appearance of NDPH may vary by nation or location for biological or cultural reasons. The same patient may be included or excluded from the diagnosis depending on which criteria were used in the research due to the shifting diagnostic criteria for NDPH (12). Although NDPH affects people of various ages, the majority of case studies featured individuals with a mean age between the twenties and the forties. Although both male and female participants are almost equally affected, past findings claimed that NDPH was more common in women. Bilateral headaches are the most frequent type; however, unilateral headaches, unilateral and bilateral headaches (on separate days), and side-shifting headaches are also unusual.

The initial recommendation for the therapy of a primary headache in adults is to rule out secondary headaches (14). Patients experience these long-term headaches because there are currently no formal standards for their treatment once it is proven that they are linked to long-term COVID (post-COVID pain syndrome). The purpose of this review is to highlight and discuss the condition of NDPH associated with COVID-19 disease through case reports. A systematic review of case reports of patients with NDPH and COVID infection will help in identifying the unusual presentations of the condition to expand the knowledge of healthcare professionals and improve patient care. It will also help in identifying patterns and similarities among cases, potential safety concerns, and early detection, investigation, and treatment strategies related to the disease.

## 2 Materials and methods

### 2.1 Eligibility criteria

A systematic review was conducted and registered in PROSPERO (Prospero registration: CRD42022354912). Post COVID-19 NDPH- phenotype related case reports were searched and identified. Studies with any kind of intervention, comparison, and outcome were not selected as the aim was to study only the case reports describing patients of Covid with NDPH.

### 2.2 Data sources and search criteria

This review followed the patient, intervention, comparison and outcome (PICO) framework (Table 1) and the Preferred Reporting Items for Systematic Reviews and Meta-Analysis (PRISMA) guidelines (15). Six data sources have been searched (on December 15th, 2022): Scopus, Web of Science, MEDLINE, Embase, MedRxiv, and Science-direct. The search terms were: “Coronavirus disease”, “Covid-19”, “Covid”, “Corona”, “SARS-Covid”, “SARS-Cov-2”, “Headache”, “New Daily Persistent Headache”, “NDPH”, “Chronic Headache”, “Case study”, “Case-report”. No restrictions were kept for the search in terms of duration. English was the language of preference. Analysing the references from pertinent papers enabled the search for more studies. A search was carried out by researchers NRD and VG.

**Table 1:** Extracted data from the original case reports along with PICO characteristics.

Author & year	Pedro Augusto Sampaio Rocha-Filho and Lara Voss, 2020 (16)	Fedelo Dino et al., 2021 (17)	Fedelo Dino et al., 2021 (17)	Adrienne C. Simmons et al., 2022 (18)	Adrienne C. Simmons et al., 2022 (18)	Adrienne C. Simmons et al., 2022 (18)	Esra Ozkan et al., 2022 (19)
(Title)							
Population	40-year-old woman	49-year-old woman	41-year-old woman	15-year-old girl	16-year-old boy	10-year-old girl	44-year-old woman
	Past comorbidity: migraine with and without aura	Past comorbidity: none, no history of headache	Past comorbidity: none, no headache	Past comorbidity: depression (treated with escitalopram)	Past comorbidity: none	Past comorbidity: None	Past comorbidity: low-frequency episodic migraine
Covid-19 symptoms onset and diagnosis procedure	Diarrhea, cough, fatigue, myalgia, anosmia, facial pain	Coughing, sneezing, asthenia, muscle aches, anosmia, and ageusia started 4 days prior to the headache’s development.	Symptoms not reported.	Symptoms not reported	Symptoms not reported	nasal congestion, Anosmia, headache rhinorrhea, and decreased appetite	Headache, fatigue, cough, and shortness of breath
	RT-PCR positive on 4th day	Real Time-PCR positive on day 3	RT-PCR positive on day 10 (at the time of admission)	RT-PCR positive on day 7	RT-PCR positive at the same time	RT-PCR positive on 2nd day	RT-PCR positive on 1st day and CT lung to rule out Pneumonia
NDPH (presentation) and other associated symptoms	Frequent (maximum days of week), mild physical activity made a bilateral, frontotemporal,pulsing headache worse.	Persistent/continuous daily headache characterized by moderate to severe occipital pain on both sides and retroorbital uneasiness, no nausea, vomiting, photophobia/phonophobia. Partial relief from taking nimesulide or acetaminophen	Persistent severe sharp, bilateral, unremitting daily frontotemporal headache with discomfort in retro-orbital area and photophobia and unresponsive to acetaminophen. Hypoasthesia on left side.	Persistent daily headaches (bitemporal pressure without radiation) pounding in nature with neck tightness.	Daily persistent bilateral frontotemporal throbbing and pulling headache with associated congestion and light-headedness on changing position.	Daily Headache (bilateral pressure behind the eyes), dizziness, photophobia and blurred vision often, lasted for one hour every afternoon, improved on rest and increased on activity.	Continuous, Pulsatile, bilateral, frontally located pressure-like feeling, nausea with severe photophobia and phonophobia
	Started on 5th day of Covid infection illness	Started before 7th day of Covid infection illness	Started before 10th day of Covid infection illness	VAS not reported.	VAS: 7/10	Started with Covid infection illness	VAS: 10/10
		VAS: 7/10	VAS: 8/10	Asthesia, pain in neck and fatigue were also present.	Started before 10th day of Covid infection illness		Started with Covid infection illness
				Started before 7th day of Covid infection illness			
Headache duration	80 days	Daily and persistent headaches even after becoming negative for Covid-19 up to day 29	The headache persisted with the same intensity and clinical features up to day 20	Seven months	Eleven months	Two months	4 months
Investigations	MRI, intracranial MRA: normal	MRI, MRA, MRV, WBC, CRP, ESR, D-dimer: all WNL	Brain CT with angiography: unremarkable, Blood tests (WBC, CRP, ESR, and D-dimer): normal	Not reported	Not reported	Not reported	Brain MRI and fundus examination: Normal
Intervention	Naproxen and Sumatriptan	IV methylprednisolone 1 mg/day and prednisolone 25 mg daily orally- 7 days	For asthenia and hypoesthesia, 0.9 mg/kg IV alteplase.	Combination of ibupfofen, caffeine, aspirin and acetaminophen along with lifestyle modifications like adequate sleep and hydration, limit caffeine and screen time.	Verapamil, physiotherapy, acupuncture and massage therapy with no improvement followed by a Prednisolone course.	Coenzyme, Q10, riboflavin, magnesium, petasin, isopetasin and buuterbur, lifestyle modifications.	Methylprednisolone, acetazolamide, subcutaneous Galcanezumab, Calcitonin Gene-Related Peptide receptor monoclonal antibody (CGRP mAB).
			For headaches: NSAIDS				
Comparison	N/A	N/A	N/A	N/A	N/A	N/A	N/A
Results	Headache improved in intensity, frequency and duration over the 80 days	Good improvement in 10 days with no side effects.	Partial improvement	No improvement	No improvement	Improved at 5-month follow-up call but reported intermittent tension-type headaches at 14 months follow-up	Improvement in both intensity and frequency within 2 days of CGRP mAB.
		VAS: 4/10	VAS: 7/10				
Outcome	A probable diagnosis of NDPH was made	Diagnosis of NDPH was made.	Diagnosis of TIA and probable NDPH was made	A probable diagnosis of NDPH was made for all patients.			A severe persistent, long-lasting post-Covid headache case successfully treated with CGRP mAB

RT-PCR, Reverse Transcription- Polymerase Chain Reaction; SARS-CoV-19, Severe Acute Respiratory Syndrome-covid-19; VAS, Visual Analog Scale; CT, Computed Tomography; MRA, Magnetic Resonance Angiography; MRV, Magnetic Resonance Venography; WBC, White Blood Count; CRP, C-Reactive Protein; ESR, Erythrocyte Sedimentation Rate; WNL, Within Normal Limits; IV, Intravenous; NSAIDS, Non-steroidal anti-inflammatory drugs; NDPH, New Daily Persistent Headache; TIA, Transient Ischemic Attack; MRI, magnetic resonance imaging; RFT, Renal Function Test.

### 2.3 Data extraction and study selection

Titles, abstracts, and full texts were analyzed by the two reviewers independently. Both reviewers mutually accepted the selection process without any kind of conflict. Both authors extracted, gathered, and compiled the data with one final report. Extracted details included the year of publication, NDPH total duration, diagnostic tests, pain severity, treatment, and conclusions (Table 1). Only case reports of patients infected with Covid-19 and persistent headaches for some duration characteristic of NDPH according to ICHD-3, even after becoming negative were included.

### 2.4 Synthesis of results

It ensures that the studies being pooled together address similar patient populations, interventions, comparisons, and outcomes. PICO characteristics, including different age groups (adults aged 26–47 years, middle-aged adults aged 48–64 years, and older adults aged 65 years or over), different treatment interventions, comparison (N/A) and outcomes like diagnosis. The information that was retrieved in this review was qualitatively synthesized. We did not perform a quantitative synthesis (e.g., meta-analysis) due to fewer case studies or reports and due to heterogeneity in multiple factors of the studies included.

### 2.5 Ethics

This analysis of published case reports is methodical. Neither the original reports nor this work contained any information about the patient's personal lives. Ethics committee permission was not required.

### 2.6 Risk of bias assessment

For the assessment of the risk of bias, a JBI critical appraisal checklist developed by Moola et al. (20), was used. The case report was considered as good quality and included in this systematic review if it satisfied 5 appraisal items out of 8. Two independent reviewers (DJ and NRD) conducted the risk of bias assessment without any disagreement.

## 3 Results

### 3.1 Selection process

**Figure 1:**
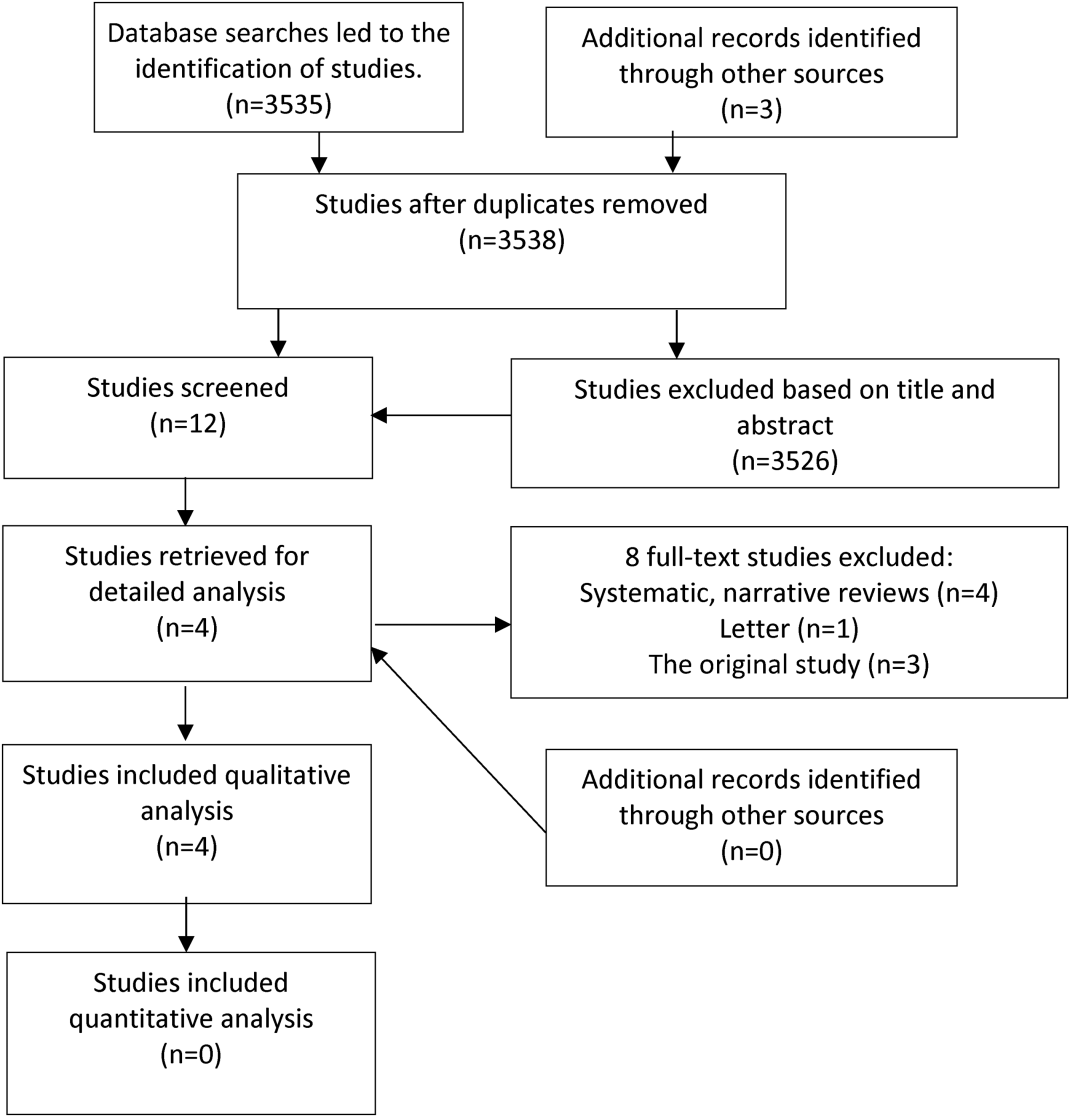
PRISMA flow diagram for selecting the studies in systematic review.

A total of 4 case reports from all the databases were found (Figure 1). Out of the total of 3,538 studies, only 12 were evaluated based on title and abstract. Lastly, eight full-text articles were excluded according to eligibility criteria, and four studies were included in the analysis, consisting of seven cases in total. The PRISMA flow diagram for the review is shown in Figure 1.

### 3.2 Evaluation of risk of bias

The JBI critical appraisal checklist for case reports was used for evaluating the risk of bias among the included studies (Table 2). Assessment criteria are described in the first row of the same table. The maximum number of cases in the case reports were described clearly demographically, chronologically, and concerning clinical features, symptoms, investigations, treatment, and results. All case reports justify the criteria for acceptability in quality for this systematic review. Only one study (18) did not describe the diagnostic tests and the important takeaways.

**Table 2:** Risk of bias assessment criteria for included studies (JBI critical appraisal checklist).

Study ID	1. Were the patient’s demographic characteristics clearly described?	2. Was the patient’s history clearly described and presented as a timeline?	3. Was the current clinical condition of the patient on presentation clearly described?	4. Were diagnostic tests or assessment methods and the results clearly described?	5. Was the intervention(s) or treatment procedure(s) clearly described?	6. Was the post-intervention clinical condition clearly described?	7. Were adverse events (harms) or unanticipated events identified and described?	8. Does the case report provide takeaway lessons?	Overall appraisal: Include Exclude Seek further info
Pedro Augusto Sampaio Rocha-Filho and Lara Voss, 2020	Yes	Yes	Yes	Yes	Yes	Yes	Yes	Yes	Include
Fedelo Dino et al, 2021	Yes	Yes	Yes	Yes	Yes	Yes	No	Yes	Include
Adrienne C. Simmons et al, 2022	Yes	Yes	Yes	No	Yes	Yes	Yes	No	Include
Esra Ozkan et al, 2022	Yes	Yes	Yes	Yes	Yes	Yes	Yes	Yes	Include

Cases of NDPH in COVID: Table 1 described the characteristics, symptoms, treatment, investigations, and results of the patients with NDPH and Covid-19. Of the seven cases (16–19), six were female only one was male. The pediatric cases were 10, 15, 16 years of age. All the adult cases were in their 40s. Cases were from Italy (*n* = 2), US (*n* = 3), Brazil (*n* = 1), and Turkey (*n* = 1). RT-PCR was used to diagnose COVID-19.

Patients had no past medical history. All had high scoring (>7) on the VAS scale except for 3 cases whose score was not reported. Cases described in the study (18), did not undergo any diagnostic imaging/blood investigations or the authors did not report them. NSAIDS and steroids were the treatment of choice in most cases. Most of the patients showed complete or partial improvement except two very young patients in the study.

## 4 Discussion

This systematic review was conducted on case reports of patients who were infected with COVID and NDPH (ICHD-III) either at the same time or near the infection. Out of the total 12 studies screened, only 4 case reports with 8 patients were selected and analyzed. Two had pre-existing headache disorders but not NDPH. One had a post-traumatic headache. Patients who responded to treatment for NDPH did not present with any special circumstances. COVID-19 is associated with regular headaches that frequently have a pattern similar to tension-type headaches or migraine phenotypes. COVID-19 could cause NDPH that is chronic, daily persistent, with a clear remembrance of the date of onset and doesn't respond to analgesics. Although the possibility of headaches lasting even after the acute stages of the COVID infection is reported, nothing is known regarding the frequency of these headache bouts and their effects. Infections with viruses may serve as a catalyst for the onset of new, daily chronic headaches (NDPH) (21).

Patients who have NDPH following a viral infection like Covid might have a different underlying etiology than those who experience NDPH following other viral infections. Only one case report on NDPH in other viral infection (Epstein Barr Virus) has been reported long time earlier (22). Her headache was not throbbing in nature and was of different nature than the headache related to Covid as seen in cases from this review. There is insufficient data to support the efficacy of any medical, surgical, or non-medical procedure that would be deemed “universally effective” for NDPH patients. By far, this is the only systematic review describing the patients with NDPH as the aftereffects of the COVID-19 viral infection.

There are other narrative reviews on NDPH but no systematic reviews of NDPH and COVID (13, 23–26). NDPH, in itself, is a rare disease, and the post-covid-NDPH phenotype is untouched. A recent review by Cheema et al. also conducted a systematic review of the main NDPH epidemiology, precipitants, phenotype, comorbidities, pathogenesis, therapy, and prognosis (12). An Italian multicentric case series to describe the characteristics of patients with NDPH following COVID infection has been recently conducted. Following COVID-19, new-onset headaches are a diverse disorder with a murky pathophysiology. With a wide range of symptoms (the New Daily Persistent Headache being the most prevalent one) and a varied response to therapy, this form of headache can become chronic and severe (27).

One patient (16) who had anosmia and facial pain along with a persistent headache could have the possibility of injury to the olfactory pathway as the SARS-CoV-2 most likely enters the central nervous system through the olfactory route (6). Viral meningitis could not be ruled out in this patient because the patient denied undergoing a spinal tap. However, the meningeal signs were absent, and this was not in favor of this possibility. This patient had a previous history of headaches, so this persistent headache may be a worsening of her previous headache. Stress is a recognized migraine trigger, and it may have made things worse. Primary headaches might often get worse from viral illnesses (28).

 NDPH was referred to as a post-infectious process (29). When SARS-CoV-2 is involved, cytokine syndrome results as a result of inflammation, which can be linked to the start of NDPH. Regarding this, a study indicated that although patients’ cerebrospinal fluid levels of tumor necrosis factor alpha were elevated, their plasma levels were normal (30). The presence of a specific pathogenic inflammatory response that can be at least partially distinguished from systemic inflammation is thought to be indicated by this. According to ICDH-3 diagnostic criteria, two individuals with NDPH were described in a different case report (17). Both patients responded well to steroid therapy, which indicates undergoing CNS inflammation as NDPH develops even if systemic inflammation is absent.

Another study (18) described only children under the age of 18. Children are less likely than adults to experience serious effects of COVID-19, even though infection and sickness are widespread among adults. The sickness primarily manifests in children as fever and cough. Fatigue, myalgia, rhinorrhoea, anosmia, and headache are other frequent complaints (31). Studies indicate that symptoms including fever and cough, which affect 10%–20% of pediatric COVID-19 cases, are more likely to be experienced by children and adolescents than headaches (32). These patients may have experienced prolonged COVID symptoms or more widespread headaches brought on by infections, but these fit the diagnosis of NDPH as defined by the International Classification of Headache Disorders. They are therefore likely cases of the NDPH phenotype, particularly when taken into account, reflecting the research demonstrated that there is a connection between any viral disease and NDPH. These cases underscore how important it is to comprehend how COVID-19 infection in children affects their neurological development.

Esra Ozkan et al. (19) presented a case of a 44-year-old woman with a severe post-COVID headache who immediately and successfully responded to CGRP mAb treatment. They gave the diagnosis of status migrainous associated with fatigue, insomnia, and memory impairments. Trigeminovascular system activation may be the cause of migraine-like symptoms in patients with no prior history of the condition, such as photophobia, phonophobia, and response to triptans (33). Instead of systemic consequences, the virus's direct infiltration was most likely what started this activation. The presence of SARS-CoV-2 proteins in the trigeminal nerve and ganglion provides evidence in favor of this theory (34). There is receptor activity modifying protein 1 (RAMP1) and protein mimics of spike protein with CGRP receptor (35). Due to the antibodies' similarity to CGRP and its receptors, this mimicry may create a long-lasting reaction in the body, which would explain the post-COVID headaches. A number of pathologic processes which causes headache, such as the dilatation of cerebral and dural blood vessels, the release of inflammatory mediators from mast cells, and the transmission of nociceptive information from intracranial blood vessels to the nervous system, are hypothesized to be influenced by CGRP. Additionally, this may explain why COVID-19 especially experiences a chronic headache as opposed to other viral systemic infections and the increased frequency of headaches. At least in some post-COVID persistent headache syndromes, a sudden cessation of the patient's chronic, severe headache after treatment with CGRP mAb may indicate activation of this pathway, providing circumstantial evidence for this pathophysiological mechanism.

Another viral infection, like the Epstein-Barr virus, has been linked to the symptoms of the NDPH, which has also been documented (30). However, more research and information are required to confirm this alleged resemblance between NDPH and long-term COVID headaches.

## 5 Conclusion

NDPH is one of the emerging relevant neurological sequelae of COVID-19 disease. Although the pathophysiological mechanism of NDPH is largely unknown, although a headache does not indicate how COVID-19 will progress, it must always be considered as a potential chronic side effect of the infection. This review has been conducted to analyze and discuss the case reports on the genesis of NDPH post-COVID-19 infection or at the time of infection. This is one of the most disturbing neurological entities occurring along with COVID, as patients described the high-level severity of the pain-causing poor quality of life. More case studies should be written and published on the same subject so that a large systematic review could be conducted. Only case reports were included in this review, as NDPH is a rare condition and epidemiological studies and systematic reviews on it are limited (36). Selection bias, heterogeneity in terms of clinical presentation, patient characteristics, treatment approaches, and outcome reported, limited generalizability, reporting bias, and publication bias are the general limitations of systematic reviews of case reports.

## Data Availability

The original contributions presented in the study are included in the article/Supplementary Material, further inquiries can be directed to the corresponding author.

## References

[1] Martelletti P Bentivegna E Luciani M Spuntarelli V. Headache as a prognostic factor for COVID-19. Time to re-evaluate. SN Compr Clin Med. (2020) 2(12):2509–10. 10.1007/s42399-020-00657-7 33263101 PMC7690334

[2] Magdy R Hussein M Ragaie C Abdel-Hamid HM Khallaf A Rizk HI Characteristics of headache attributed to COVID-19 infection and predictors of its frequency and intensity: a cross sectional study. Cephalalgia. (2020) 40(13):1422–31. 10.1177/0333102420965140 33146038 PMC7645600

[3] Howard LM Garguilo K Gillon J Seegmiller AC Schmitz JE Webber SA Characteristics and clinical features of SARS-CoV-2 infections among ambulatory and hospitalized children and adolescents in an integrated health care system in Tennessee. medRxiv [Preprint]. (2020). 10.1101/2020.10.08.20208751 33083809 PMC7574258

[4] Prakash S Shah ND. Post-infectious new daily persistent headache may respond to intravenous methylprednisolone. J Headache Pain. (2010) 11(1):59–66. 10.1007/s10194-009-0171-x 19936615 PMC3452180

[5] Headache Classification Committee of the International Headache Society. The international classification of headache disorders: 2nd edition. Cephalalgia. (2004) 24:9–160. 10.1111/j.1468-2982.2003.00824.x 14979299

[6] Classification internationale des céphalées (ICHD-3β)11Headache Classification Committee of the International Headache Society. The international classification of headache disorders. Edition-3beta. Cephalalgia. (2013) 33:629–809. 10.1177/0333102413485658 23771276

[7] Tyagi A. New daily persistent headache. Ann Indian Acad Neurol. (2012) 15(Suppl 1):S62–5. 10.4103/0972-2327.100011 23024565 PMC3444222

[8] Uniyal R Paliwal VK Tripathi A. Psychiatric comorbidity in new daily persistent headache: a cross-sectional study. Eur J Pain. (2017) 21(6):1031–8. 10.1002/ejp.1000 28146324

[9] Castillo J Muñoz P Guitera V Pascual J. Kaplan award 1998. Epidemiology of chronic daily headache in the general population. Headache. (1999) 39(3):190–6. 10.1046/j.1526-4610.1999.3903190.x 15613213

[10] Singh AK Shukla R Trivedi JK Singh D. Association of psychiatric co-morbidity and efficacy of treatment in chronic daily headache in Indian population. J Neurosci Rural Pract. (2013) 4(2):132–9. 10.4103/0976-3147.112736 23914085 PMC3724287

[11] Baron EP Rothner AD. New daily persistent headache in children and adolescents. Curr Neurol Neurosci Rep. (2010) 10(2):127–32. 10.1007/s11910-010-0097-3 20425237

[12] Cheema S Mehta D Ray JC Hutton EJ Matharu MS. New daily persistent headache: a systematic review and meta-analysis. Cephalalgia. (2023) 43(5):3331024231168089. 10.1177/03331024231168089 37032616

[13] Arca KN Starling AJ. Treatment-Refractory Headache in the Setting of COVID-19 Pneumonia: Migraine or Meningoencephalitis?. 10.1007/s42399-020-00369-y PMC7314569 32838146

[14] Becker WJ Findlay T Moga C Scott NA Harstall C Taenzer P. Guideline for primary care management of headache in adults. Can Fam Physician. (2015) 61(8):670–9. 26273080 PMC4541429

[15] Moher D Shamseer L Clarke M Ghersi D Liberati A Petticrew M Preferred reporting items for systematic review and meta-analysis protocols (PRISMA-P) 2015 statement. Syst Rev. (2015) 4(1):1. 10.1186/2046-4053-4-1 25554246 PMC4320440

[16] Rocha-Filho S Voss L. Persistent headache and persistent anosmia associated with COVID-19. Headache. (2020) 60(8):1797–9. 10.1111/head.13941 32790179 PMC7436496

[17] Dono F Consoli S Evangelista G D’Apolito M Russo M Carrarini C New daily persistent headache after SARS-CoV-2 infection: a report of two cases. Neurol Sci. (2021) 42(10):3965–8. 10.1007/s10072-021-05444-3 34264414 PMC8280630

[18] Simmons AC Bonner A Giel A Pezzano A Rothner AD. Probable new daily persistent headache after COVID-19 in children and adolescents. Pediatr Neurol. (2022) 132:1–3. 10.1016/j.pediatrneurol.2022.04.009 35598584 PMC9045862

[19] Özkan E Celebi Ö Keskin Ö Gursoy A Gürsoy-Özdemir Y. Is persistent post-COVID headache associated with protein-protein interactions between antibodies against viral spike protein and CGRP receptor?: a case report. Front Pain Res (Lausanne). (2022) 3:858709. 10.3389/fpain.2022.858709 35434707 PMC9011137

[20] Moola S Munn Z Tufanaru C Aromataris E Sears K Sfetc R Chapter 7: systematic reviews of etiology and risk. In: Aromataris E Munn Z, editors. JBI Manual for Evidence Synthesis. Australia: JBI (2020).

[21] Rozen TD. New daily persistent headache: an update. Curr Pain Headache Rep. (2014) 18(7):431. 10.1007/s11916-014-0431-6 24820732

[22] Hamada T Ohshima K Ide Y Sakato S Takamori M. A case of new daily persistent headache with elevated antibodies to Epstein-Barr virus. Jpn J Med. (1991) 30(2):161–3. 10.2169/internalmedicine1962.30.161 1650858

[23] Riddle EJ Smith JH. New daily persistent headache: a diagnostic and therapeutic odyssey. Curr Neurol Neurosci Rep. (2019) 19(5):21. 10.1007/s11910-019-0936-9 30888529

[24] Martelletti P Bentivegna E Spuntarelli V Luciani M. Long-COVID headache. SN Compr Clin Med. (2021) 3(8):1704–6. 10.1007/s42399-021-00964-7 34036244 PMC8136258

[25] Fiala K Martens J Abd-Elsayed A. Post-COVID pain syndromes. Curr Pain Headache Rep. (2022) 26:379–83. 10.1007/s11916-022-01038-6 35267156 PMC8907389

[26] Membrilla JA Caronna E Trigo-Lopez J. Persistent headache after COVID-19: pathophysiology, clinic and treatment. Neurol Perspect. (2021) 1:S31–36. 10.1016/j.neurop.2021.10.003 38620971 PMC8669731

[27] Torrente A Alonge P Di Stefano V Baschi R Ornello R Correnti E New-onset headache following COVID-19: an Italian multicentre case series. J Neurol Sci. (2023) 446(120591):120591. 10.1016/j.jns.2023.120591 36807975 PMC9931424

[28] Rocha-Filho S Torres PA Ramos R. HIV and headache: a cross-sectional study. Headache. (2017) 57:1545–50. 10.1111/head.13183 28905376

[29] Mack KJ. What incites new daily persistent headache in children? Pediatr Neurol. (2004) 31(2):122–5. 10.1016/j.pediatrneurol.2004.02.006 15301832

[30] Rozen T Swidan SZ. Elevation of CSF tumor necrosis factor *α* levels in new daily persistent headache and treatment refractory chronic migraine. Headache. (2007) 47:1050–5. 10.1111/j.1526-4610.2006.00722.x 17635596

[31] She J Liu L Liu W. COVID-19 epidemic: disease characteristics in children. J Med Virol. (2020) 92(7):747–54. 10.1002/jmv.25807 32232980 PMC7228385

[32] Viner RM Ward JL Hudson LD Ashe M Patel SV Hargreaves D Systematic review of reviews of symptoms and signs of COVID-19 in children and adolescents. Arch Dis Child. (2020) 106(8):802–7. 10.1136/archdischild-2020-320972 33334728

[33] Caronna E Alpuente A Torres-Ferrus M Pozo-Rosich P. Toward a better understanding of persistent headache after mild COVID-19: three migraine-like yet distinct scenarios. Headache. (2021) 61(8):1277–80. 10.1111/head.14197 34363619 PMC8444902

[34] Meinhardt J Radke J Dittmayer C. Olfactory transmucosal SARS- CoV-2 invasion as a port of central nervous system entry in individuals with COVID-19. Nat Neurosci. (2021) 24:168–75. 10.1038/s41593-020-00758-5 33257876

[35] Kanduc D. From anti-SARS-CoV-2 immune response to the cytokine storm via molecular mimicry. Antibodies (Basel). (2021) 10(4):36. 10.3390/antib10040036 34698069 PMC8544210

[36] Peng K-P Wang S-J. Update of new daily persistent headache. Curr Pain Headache Rep. (2022) 26(1):79–84. 10.1007/s11916-022-01005-1 35076874 PMC8787738

